# When metagenomic next-generation sequencing detects *Nocardia*: from clinical interpretation to immune-based clinical management

**DOI:** 10.3389/fcimb.2026.1911562

**Published:** 2026-09-18

**Authors:** Ping Li, Xiao Yang, Yuxia Chen, Lili Wang, Qiqi Cui, Zhihong Wu, Fenglin Cao

**Affiliations:** 1 Department of Clinical Laboratory, Qilu Hospital of Shandong University, Jinan, Shandong, China; 2 School of Nursing and Rehabilitation, Shandong University, Jinan, Shandong, China; 3 Jinan AXZE Medical Test Laboratory, Jinan, Shandong, China; 4 Department of Traditional Chinese Medicine, Qilu Hospital of Shandong University, Jinan, Shandong, China

**Keywords:** diagnosis, immune status, metagenomic next-generation sequencing (mNGS), nocardiosis, optimal threshold value

## Abstract

**Background:**

Metagenomic next-generation sequencing (mNGS) increasingly detects *Nocardia*, but interpreting positive results remains challenging. We aimed to identify host factors and read thresholds that distinguish nocardiosis (ND) from colonization/contamination, and to characterize clinical features linked to immune status.

**Methods:**

A retrospective analysis from 74 patients at Qilu Hospital of Shandong University from April 2022 to October 2025 was conducted. We assessed mNGS for distinguishing *Nocardia* infection from colonization or contamination, identified risk factors for nocardiosis, and compared patients’ clinical features, imaging findings, and antimicrobial adjustments.

**Results:**

A total of 56 patients with ND and 18 non-ND patients were included. Multivariate logistic regression identified farming exposure, bronchiectasis, and immunocompromised status were significantly associated with ND. An estimated read per million (RPM) thresholds of 56 could distinguish *Nocardia* infection from colonization/contamination (AUC 0.774; 95% CI, 0.654 – 0.895), with a higher sensitivity than traditional tests (*P* < 0.001). In ND group, immunosuppressed patients (ISPs) (42.9%) had more pulmonary interstitial fibrosis (29.17% vs. 6.25%, *P* = 0.03), lymphadenopathy (62.50% vs. 40.63%, *P* = 0.046) and fungal co-infections (54.17% vs. 18.75%, *P* = 0.006), while bronchial wall thickening was more common in immunocompetent patients (ICPs) (31.25% vs. 4.17%, *P* = 0.019). *Nocardia* was the sole pathogen in 42.9% of true infection cases, and mNGS led to a change in anti-*Nocardia* management in 87.5% of patients.

**Conclusion:**

Our study indicated mNGS is a useful tool for distinguishing *Nocardia* infection from colonization/contamination, and immune-based phenotyping may guide individualized management strategies for patients with nocardiosis.

## Introduction

Nocardiosis is a rare but potentially life-threatening infection caused by *Nocardia*, a ubiquitous environmental actinomycete (Kandi, 2015). The definitive diagnosis of nocardiosis depends on isolation and culture of *Nocardia* from suspected sites, which usually takes 3–5 days and the sensitivity may be decreased by prior antibiotics usage (Weng et al., 2020). Moreover, the clinical presentation of nocardiosis is often subacute to chronic in onset, with nonspecific symptoms (Brown-Elliott et al., 2006; Restrepo and Clark, 2019). Thus, *Nocardia* is prone to being missed, delayed or misdiagnosed in the clinic, resulting in inappropriate empirical therapy and poor outcomes.

In recent years, the incidence of nocardiosis has increased, which is partly attributed to the improvement of diagnostic methods, especially the application of mNGS (Ding et al., 2021). As a complementary diagnostic modality to conventional clinical testing, mNGS demonstrates high diagnostic utility in nocardiosis, and it could support clinical decision-making by reducing turnaround time (Miao et al., 2018; Weng et al., 2020). However, interpreting a positive mNGS result of *Nocardia* remains challenging due to the difficulty of DNA extraction from the thick polysaccharide cell wall, as well as contamination of clinical specimens and colonization of the respiratory tract (Restrepo and Clark, 2019; Liang et al., 2023). Therefore, it is urgently necessary to establish pathogen-specific read thresholds to scientifically evaluate the clinical utility of mNGS in distinguishing *Nocardia* infection from non-significant detection.

*Nocardia* primarily causes infections in immunocompromised hosts, but it can also lead to life-threatening disease in immunocompetent patients (Liang et al., 2023). However, large comparative studies stratifying patients by immune status are lacking, with most evidence limited to case reports or small series (Verfaillie et al., 2010; Chen et al., 2014; Coussement et al., 2016). Therefore, a systematic analysis of a larger nocardiosis cohort stratified by immune status would improve clinical understanding and enable more personalized management.

In this study, we aimed to establish a practical framework for interpreting positive mNGS results of *Nocardia* by: (1) identifying host factors and an mNGS RPM threshold that differentiate true infection from colonization/contamination; and (2) characterizing the distinct clinical, radiological, and microbiological features of confirmed nocardiosis according to immune status, to guide immune-based stratification.

## Materials and methods

### Study design and patient recruitment

We recruited patients with *Nocardia* species detected by mNGS in clinical specimens at Qilu Hospital of Shandong University from April 2022 to October 2025. Patients were eligible for enrollment if they met all the following criteria: (1) RPM reads of *Nocardia* > 1; (2) complete medical records including conventional microbiological tests (smear, culture) performed within 2 hours of mNGS sampling. (3) age ≥ 18 years. Exclusion criteria: (1) lack of concurrent conventional tests; (2) incomplete clinical data.

Immunocompromised conditions were defined according to the following criteria (Hill, 2020): (1) hematologic malignancies; (2) solid tumors receiving cytotoxic chemotherapy within 28 days; (3) hematopoietic stem cell transplantation or solid organ transplantation; (4) active rheumatic/autoimmune disease requiring immunosuppressants; (5) long-term use of corticosteroids (0.3 mg/kg/day of prednisone equivalent for 3 weeks). Patients meeting multiple criteria were counted once.

The demographic characteristics, underlying diseases, patients’ immune status, laboratory test results, CT of the chest, treatment, and outcomes were collected in the hospital information systems. All the data were reviewed by two investigators independently to verify data accuracy.

### Definitions of ND and non-ND

A patient was classified as ND (true nocardiosis) if all of the following criteria were met: (1) mNGS positive for *Nocardia* (RPM > 1); (2) corresponding clinical symptoms of infection (e.g., fever, cough, dyspnea); (3) either culture/smear positive, or radiological improvement after anti-*Nocardia* therapy; and (4) no alternative pathogen fully explaining the clinical syndrome.

A patient was classified as non-ND (colonization/contamination) if any of the following applied: (1) clinical improvement without anti-*Nocardia* therapy; (2) an alternative pathogen identified as the sole cause of illness; (3) asymptomatic detection with no evidence of nocardiosis during follow-up. For all non-ND patients, clinical follow-up data were systematically collected through electronic medical records and outpatient visit documentation to exclude delayed manifestations of nocardiosis.

The classification was performed independently by two infectious disease specialists based on all available clinical, radiological, and microbiological data; disagreements were resolved by consensus.

### Specimen collection

Samples from the infected location were collected using aseptic techniques when clinicians suspect a patient has an infection caused by pathogenic microorganisms, but have not yet found etiological evidence. A portion of specimens was sent for culture and smear within 2 h, while the remaining specimens were stored at 4 °C for mNGS analysis within 12h.

### Conventional microbiological tests

The clinical specimens were prepared for smear (Gram staining and modified acid-fast staining), and cultured on different agar plates simultaneously. Blood samples were inoculated into aerobic blood culture bottles and incubated in an automated blood culture monitoring system. The processed BALF specimens were inoculated onto blood agar, Mueller-Hinton/Mac conkey (MH/MAC) agar and chocolate agar, spreading evenly, and incubated at 35°C for 5–7 days in aerobic conditions.

*Nocardia* isolates were identified via matrix-assisted laser desorption/ionization time-of-flight mass spectrometry (MALDI-TOF-MS) using a MALDI Biotyper (Bruker Daltonics). A MALDI-TOF-MS scores between 1.7 to 2.0 represented genus-level identification, while a score of higher to 2.0 represented identification at species level. Anything less than 1.7 indicated unreliable identification.

### mNGS and analysis

The process of mNGS analysis included Deoxyribonucleic acid (DNA) extraction, library construction, high-throughput sequencing, and bioinformatics analysis in the BGISEQ-200 platform. By comparing the nucleic acid sequences of microorganisms with the known bacterial sequences stored in the database, the types of microorganisms can be identified. Various parameters of the sequencing platforms include the number of mapped reads (at the species and genus levels), abundance (at the species and genus levels), depth, and coverage. These factors are used to eliminate any interference that other microorganisms may cause. We normalized the sequencing reads RPM to eliminate the errors caused by various sequencing depths among samples. To minimize and monitor contamination, the following measures were implemented: (1) negative controls (nuclease-free water) were processed in parallel through the entire workflow; (2) reagent blanks were included to monitor kit-derived contamination; (3) all procedures were performed in a dedicated pre-amplification area with strict separation from post-amplification products; (4) negative controls were sequenced alongside patient samples in each batch; (5) a microbial background database was established to filter environmental contaminants, and reads detected in negative controls were subtracted from patient sample reads; and (6) all ambiguous signals were reviewed by a multidisciplinary team. The mNGS test was conducted according to the manufacturer’s protocol, and detailed information can be found in **Supplementary Appendix 2**.

### Statistical analysis

Continuous variables were expressed as median (IQR) or the mean ± standard deviation (SD), and compared with T-test or Mann-Whitney U test. Categorical variables, shown as frequencies and percentages [n (%)], were compared by χ2 test or Fisher’s exact test. Univariate and multivariate logistic regression models were used to explore the risk factors associated with ND diagnosis.

To determine the optimal RPM threshold for distinguishing *Nocardia* infection from colonization/contamination, ROC curves were constructed and the Youden index (sensitivity + specificity − 1) was used to select the optimal cutoff value. Sensitivity, specificity, positive predictive value (PPV), and negative predictive value (NPV) were calculated with 95% confidence intervals (CIs) using the Wilson score method to account for binomial proportions. MedCalc Statistical Software (Version 23.6.3) was used for CI calculations. Diagnostic performance was compared using McNemar’s test for paired proportions.

To assess the robustness of the ROC-derived optimal RPM threshold, bootstrap internal validation was performed with 1,000 resampling iterations, from which the median optimal cutoff and its 95% bootstrap CI were derived. Model generalizability was evaluated by comparing the AUC and Youden index between in-bag and out-of-bag samples. Analyses were conducted using the cutpointr package (v1.1.2) in R (v4.5.2). All statistical analyses were performed using SPSS (Version 25.0) and GraphPad Prism (Version 10.1.2), and p < 0.05 was considered statistically significant.

## Results

### Baseline characteristics and sample classification

A total of 56 patients with ND and 18 non-ND patients were included in this study. The baseline characteristics of all eligible patients are presented in **Table 1**. There were no significant differences between the ND group and non-ND groups in terms of age, gender, length of hospital stays and symptoms. However, the proportion of farming exposure was significantly higher in the ND group than in the non-ND group (60.71% vs. 33.33%, *P* = 0.043). Meanwhile, we observed that patients with bronchiectasis (37.50% vs. 11.11%, *P* = 0.043) and rheumatic immune diseases (23.21% vs. 0.00%, *P* = 0.03) were more likely to have *Nocardia* infection. As expected, 42.86% of the ND patients had immunocompromised conditions, which was far less common in the non-ND cohort (42.86% vs. 11.11%, *P* = 0.03).

**Table 1:** Baseline data of patients in ND and non-ND groups.

Characteristics	ND (n=56)	non-ND (n=18)	*P* value
**Male, n (%)**	30 (53.57%)	11 (61.11%)	0.576
**Age (year)**	62.52 ± 13.34	59.28 ± 10.88	0.353
**Farming exposure, n (%)**	34 (60.71%)	6 (33.33%)	0.043^*^
**Hospital days**	12 (9-20)	13.5 (8-19.5)	0.915
Symptoms, n (%)			
Fever (body temperature > 37.1°C)	33 (58.93%)	6 (33.33%)	0.058
Cough and expectoration	45 (80.36%)	11 (61.11%)	0.098
Chest pain	5 (8.93%)	2 (11.11%)	1.000
Hemoptysis	7 (12.50%)	1 (5.56%)	0.670
Dyspnea	26 (46.43%)	8 (44.44%)	0.883
Underlying pulmonary diseases, n (%)			
COPD	7 (12.50%)	0 (0.00%)	0.184
Bronchiectasis	21 (37.50%)	2 (11.11%)	0.043^*^
Pulmonary interstitial fibrosis	9 (16.07%)	0 (0.00%)	0.102
Bronchial asthma	5 (8.93%)	2 (11.11%)	1.000
Extrapulmonary diseases, n (%)			
Hypertension	17 (30.36%)	3 (16.67%)	0.405
Diabetes mellitus	7 (12.50%)	5 (27.78%)	0.126
Rheumatic immune diseases	13 (23.21%)	0 (0.00%)	0.030^*^
Hematologic malignancy	1 (1.79%)	1 (5.56%)	0.430
Active solid tumor	6 (10.71%)	0 (0.00%)	0.325
**Immunocompromised, n (%)**	24 (42.86%)	2 (11.11%)	0.030^*^
Radiological findings, n (%)			
Ground-glass opacities	44 (78.57%)	13 (72.22%)	0.402
Nodules	28 (50.00%)	7 (38.89%)	0.341
Air bronchial sign	6 (10.71%)	1 (5.56%)	0.672
Cavitation sign	8 (14.29%)	3 (16.67%)	1.000
Pleural thickening	14 (25.00%)	2 (11.11%)	0.326
Pleural effusion	23 (41.07%)	6 (33.33%)	0.488
Lymphadenopathy	28 (50.00%)	7 (38.89%)	0.341
Laboratory findings			
WBC (10^9^/L)	11.02 (6.96-15.02)	6.66 (4.84-9.34)	0.003^**^
Neutrophils (10^9^/L)	8.69 (5.12-13.36)	4.72 (3.12-7.96)	0.005^**^
Lymphocytes (10^6^/L)	0.99 (0.67-1.50)	1.06 (0.70-1.51)	0.632
ESR (mm/h)	64.28 ± 33.77	39.71 ± 34.41	0.024^*^
CRP (mg/l)	59.99 (9.36-122.45)	15.92 (3.33-53.81)	0.041^*^
PCT (ng/ml)	0.13 (0.06-0.37)	0.31 (0.05-1.73)	0.298
Outcome, n (%)			
28 days-mortality	7 (12.50%)	1 (5.56%)	0.670

ND, Nocardiosis Disease; COPD, chronic obstructive pulmonary disease; ESR, erythrocyte sedimentation rate; CRP, C-response protein; PCT, procalcitonin.

^*^ *P* < 0.05; ^**^P < 0.01.

As shown in **Table 1**, the white blood cell count (*P* = 0.003), neutrophil count (*P* = 0.005), Erythrocyte sedimentation rate (ESR, *P* = 0.024) and C-reactive protein levels (CRP, *P* = 0.041) were significantly higher in the ND group than in the non-ND group. The most common radiological findings on chest CT in ND patients were ground-glass opacities (78.57%), nodules (50.0%), and mediastinal or hilar lymphadenopathy (50.0%).

Among the 18 patients initially classified as non-ND, 17 had assessable follow-up data (One patient died of heart failure 10 days after discharge, and one was lost to follow-up). The median follow-up duration for the remaining 16 patients was 153.5 days (IQR: 58–412 days; range: 27–812 days). During this period, none of the patients developed clinical or radiological evidence of nocardiosis, supporting the initial classification of colonization/contamination (**Supplementary Appendix 1**).

The abundance of *Nocardia* sequences detected by mNGS was significantly higher in the ND group than in the non-ND group (*P* = 0.0003) (**Figure 1A**). The most frequent sample type observed was BALF, followed by sputum (**Figure 1B**). In the ND group, *Nocardia farcinica* was the most prevalent species (30.36%, 17/56), followed by *N. cyriacigeorgica* (28.57%, 16/56) and *N. abscessus* (12.50%, 7/56). In the non-ND group, the predominant species were *N. farcinica* (33.33%, 6/18), *Nocardia* spp. (22.22%, 4/18), *N. cyriacigeorgica* (16.67%, 3/18), and *N. gipuzkoensis* (16.67%, 3/18) (**Figure 1C**).

**Figure 1:**
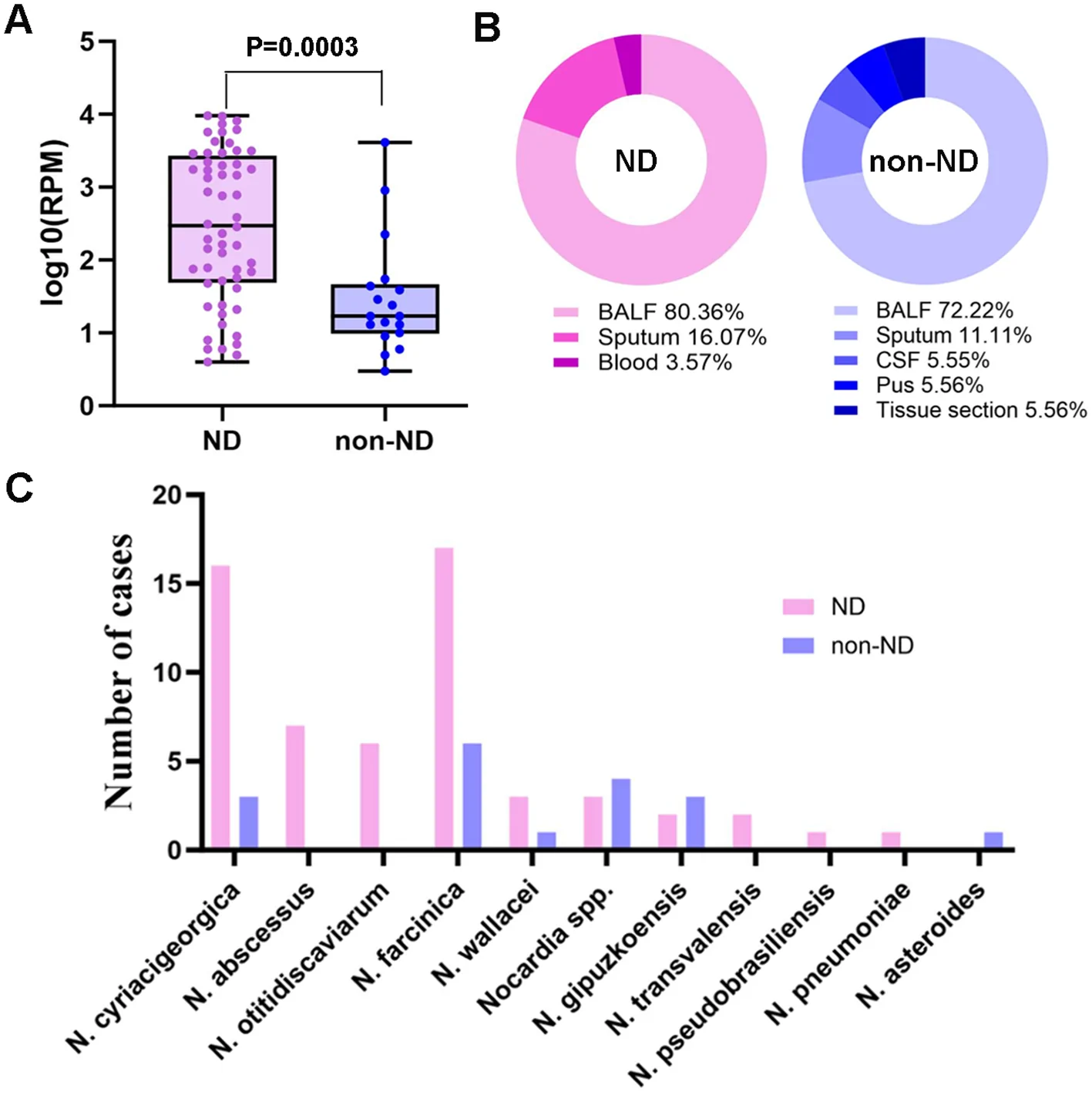
Distribution and abundance of *Nocardia* species in the ND and non-ND groups. **(A)** Differences in mNGS RPM of *Nocardia* between the ND and non-ND groups. **(B)** Distribution of clinical specimen types from which *Nocardia* was detected by mNGS in the ND and non-ND groups. **(C)** Comparison of *Nocardia* species between the ND and non-ND groups.

### Factors associated with ND diagnosis

Univariate logistic regression identified three factors significantly associated with a diagnosis of ND: farming exposure (OR = 5.489; 95% CI, 1.697–17.756; P = 0.004), bronchiectasis (OR = 4.800; 95% CI, 1.002–22.988; *P* = 0.050), and immunocompromised status (OR = 6.000; 95% CI, 1.258–28.620; *P* = 0.025) (**Table 2**). These variables were subsequently entered into a multivariate logistic regression model. After adjustment for potential confounders, all three remained significantly associated with ND: farming exposure (OR = 5.826; 95% CI, 1.576–21.537; *P* = 0.008), bronchiectasis (OR = 7.316; 95% CI, 1.317–40.653; *P* = 0.023), and immunocompromised status (OR = 8.698; 95% CI, 1.593–47.493; *P* = 0.013).

**Table 2:** Factors associated with ND diagnosis.

Variable	Univariate analysis		Multivariate analysis	
	OR (95%CI)	*P* value	OR (95%CI)	*P* value
Farming exposure	5.489 (1.697-17.756)	0.004^**^	5.826 (1.576-21.537)	0.008^**^
Bronchiectasis	4.800 (1.002-22.988)	0.050	7.316 (1.317-40.653)	0.023^*^
Immunocompromised	6.000(1.258-28.620)	0.025^*^	8.698 (1.593-47.493)	0.013^*^

^*^ *P* < 0.05; ^**^P < 0.01.

To explore the potential additive value of radiological features, we performed an exploratory multivariate logistic regression model incorporating lymphadenopathy and bronchial wall thickening together with the three host factors identified in the primary analysis. In this exploratory multivariate model, neither radiological parameter reached statistical significance (lymphadenopathy: OR = 1.139; 95% CI, 0.288–4.506; *P* = 0.853; bronchial wall thickening: OR = 0.760; 95% CI, 0.112–5.180; *P* = 0.779). The three host factors remained significant independent predictors: farming exposure (OR = 8.804; 95% CI, 1.563–21.559; *P* = 0.009), bronchiectasis (OR = 8.362; 95% CI, 1.228–56.930; *P* = 0.030), and immunocompromised status (OR = 7.322; 95% CI, 1.285–41.740; *P* = 0.025). These results suggest that radiological features do not provide independent predictive value beyond host factors.

### Diagnostic efficacy of mNGS for *Nocardia* infection

To determine the optimal RPM threshold distinguishing *Nocardia* infection from colonization/contamination, we constructed a ROC curve using the *Nocardia* RPM of mNGS from enrolled patients. The area under curve (AUC) was 0.774 (95% CI, 0.654–0.895), and the estimated optimal cut-off value was 56 (**Figure 2A**). Using RPM ≥ 56 as the diagnostic criterion for *Nocardia* infection, mNGS achieved a sensitivity of 73.21% (95% CI, 59.70%–84.17%), specificity of 83.33% (95% CI, 58.58%–96.42%), PPV of 93.18% (95% CI, 82.78%–97.49%) and NPV of 50% (95% CI, 38.23%–61.77%) (**Figure 2B**). In comparison, culture had a sensitivity of 25% (95% CI, 14.39%–38.37%), specificity of 100% (95% CI, 81.47%–100.00%), PPV of 100% (95% CI, 76.84%–100.00%) and NPV of 30% (95% CI, 26.92%–33.27%). The sensitivity of mNGS was superior to that of traditional microbial tests, as demonstrated by comparisons with culture (*P* < 0.001) and smear (*P* < 0.001), while specificities did not differ significantly. Furthermore, culture exhibited significantly higher sensitivity than smear (*P* = 0.039) (**Figures 2C, D**).

**Figure 2:**
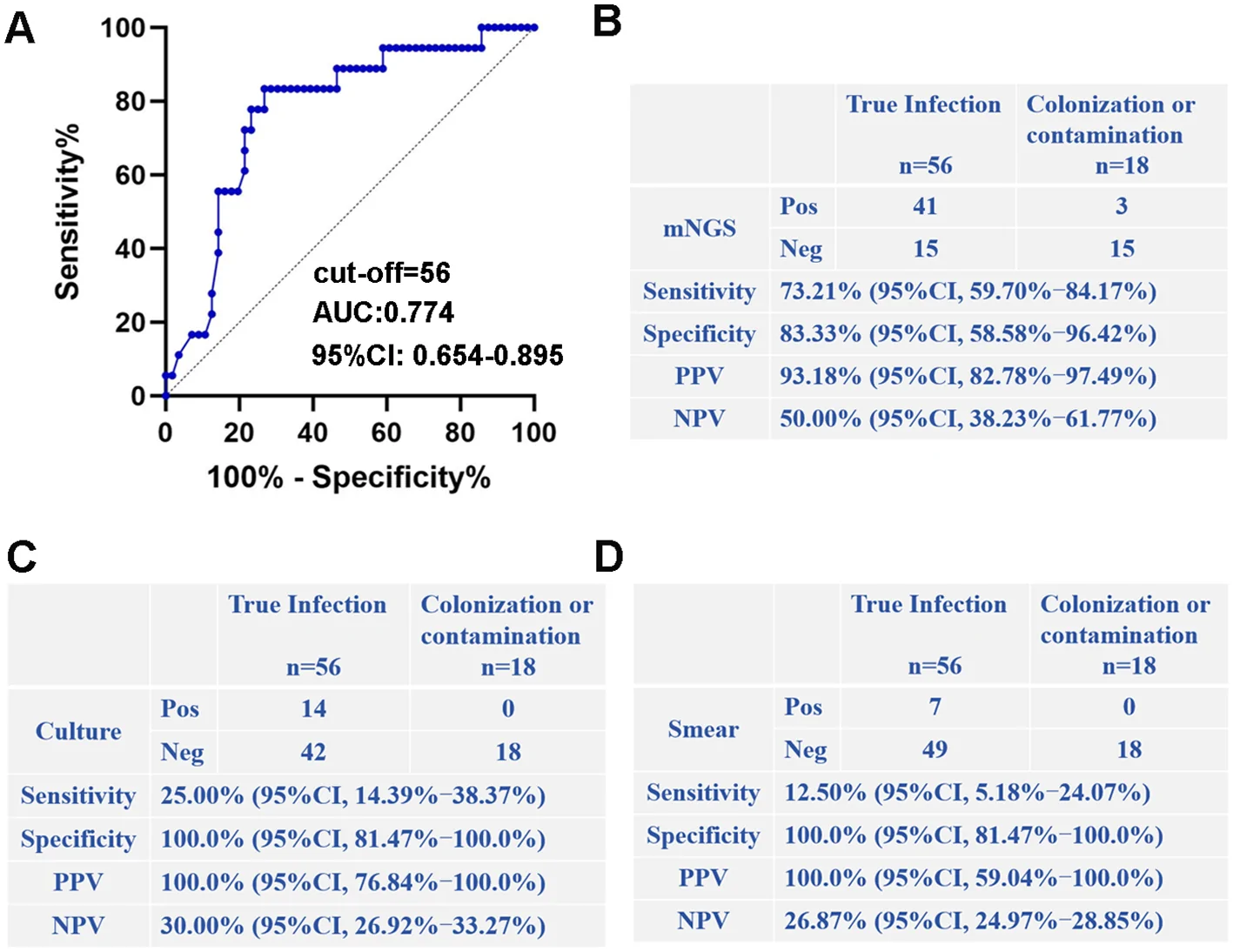
Diagnostic performance of mNGS, Culture, and Smear for distinguishing *Nocardia* infection from colonization/contamination. **(A)** ROC curve of mNGS RPM values for distinguishing *Nocardia* infection from colonization/contamination. The AUC was 0.774 (95% CI: 0.654–0.895); the optimal cutoff was RPM = 56. **(B–D)** Diagnostic performance of mNGS **(B)**, Culture **(C)**, and Smear **(D)** for differentiating *Nocardia* infection from colonization/contamination.

A subgroup ROC analysis was performed exclusively in immunocompetent individuals, yielding an AUC of 0.845 (95% CI: 0.722–0.968) (**Figure 3A**). Applying the overall-cohort threshold (RPM ≥ 56) to the subgroup gave a sensitivity of 81.25% (95% CI, 63.56%–92.79%) and specificity of 81.25% (95% CI, 54.35%–95.95%), which were comparable to those obtained with the immunocompetent-specific threshold (RPM ≥ 46: sensitivity 87.5% (95% CI, 71.01%–96.49%), specificity 75.0% [(95% CI, 47.62%–92.73%)] (**Figures 3B, C**), suggesting that the overall threshold may be reasonably applied to immunocompetent patients.

**Figure 3:**
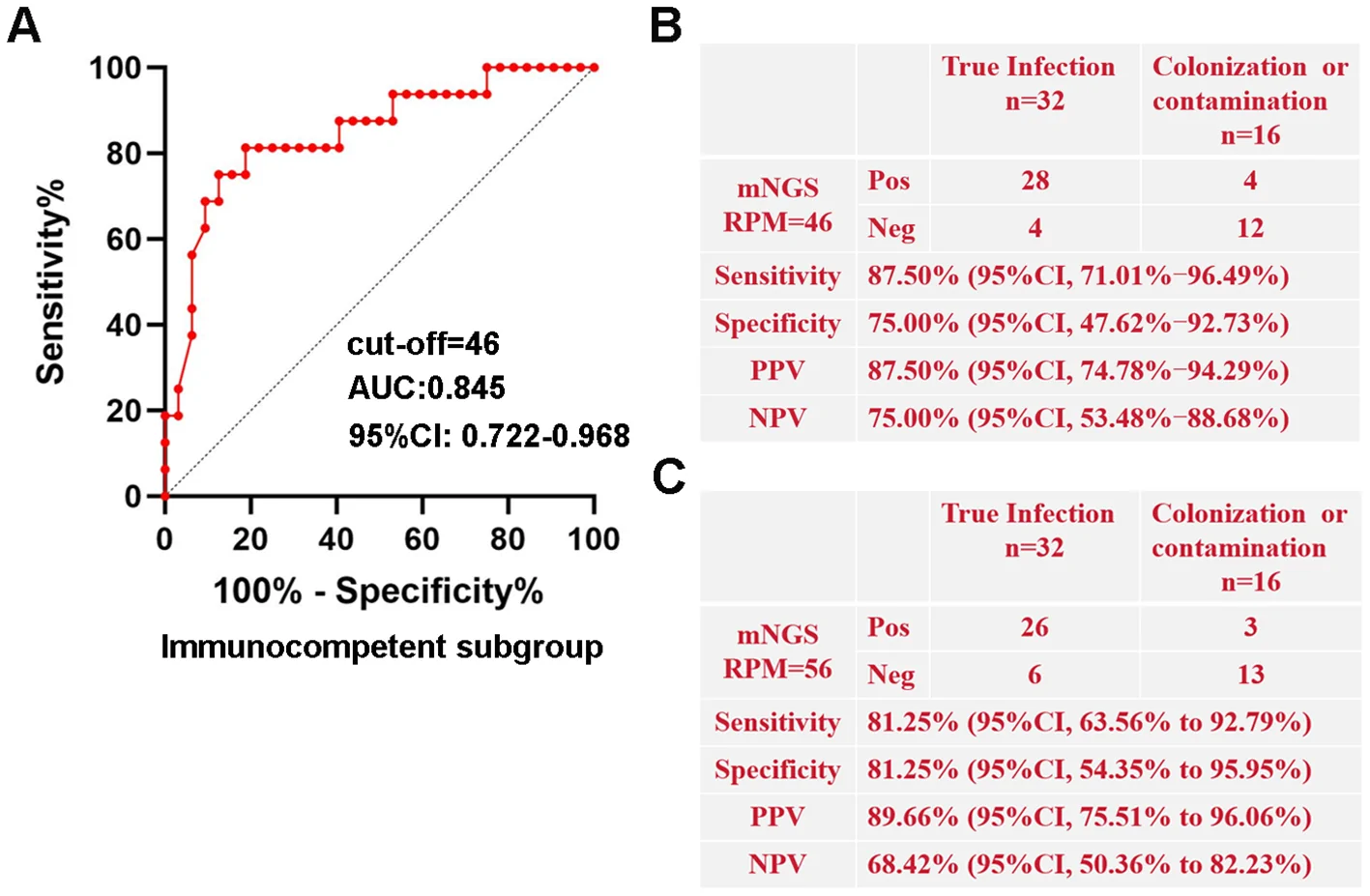
Diagnostic performance of mNGS for distinguishing *Nocardia* infection from colonization/contamination in immunocompetent patients (ICPs). **(A)** ROC curve of mNGS RPM values for distinguishing *Nocardia* infection from colonization/contamination in the ICP subgroup. The AUC was 0.845 (95% CI: 0.722–0.968); the optimal cutoff was RPM = 46. **(B)** Diagnostic performance of mNGS using the ICP-specific optimal cutoff of RPM = 46 in the ICP subgroup. **(C)** Diagnostic performance of mNGS using the overall-cohort cutoff of RPM = 56 applied to the ICP subgroup.

To assess the robustness of the RPM threshold derived from the ROC analysis, we performed internal validation using bootstrap resampling with 1,000 iterations. Bootstrap validation showed that the median optimal RPM cutoff was 57 (95% CI: 21–285), closely approximating the original threshold of 56. The mean in-bag and out-of-bag AUC values were both 0.774, indicating good generalizability without overfitting. The mean in-bag and out-of-bag Youden indices were 0.584 and 0.472, respectively.

### Characteristics of ND patients with different immune status

We further analyzed the clinical characteristics of ND patients according to immune statuses. According to **Table 3**, the incidence of pulmonary interstitial fibrosis and rheumatic immune diseases were higher in the ISPs group than in the ICPs group (29.17% vs. 6.25%, *P* = 0.03;54.17% vs. 0.00%, *P* < 0.001, respectively). Retrospective analysis of radiological images showed that lymphadenopathy was more common in ISPs patients (62.50% vs. 40.63%, *P* = 0.046), whereas bronchial wall thickening was more frequently observed in ICPs patients (31.25% vs. 4.17%, *P* = 0.019).

**Table 3:** The comparison of characteristics between ICPs and ISPs in ND group.

Characteristics	ICPs(n=32)	ISPs (n=24)	*P* value
**Male, n (%)**	15 (46.88%)	15 (62.50%)	0.246
**Age (year)**	62.91 ± 14.08	62 ± 12.57	0.804
**Hospital days**	12(8-19.75)	14.5(9-21)	0.518
Symptoms, n (%)			
Fever (body temperature > 37.1°C)	20 (62.50%)	13 (54.17%)	0.530
Cough and expectoration	27 (84.38%)	18 (75.00%)	0.382
Chest pain	2 (6.25%)	3 (12.50%)	0.735
Hemoptysis	6 (18.75%)	1 (4.17%)	0.219
Dyspnea	16 (50.00%)	10 (41.67%)	0.536
Underlying diseases, n (%)			
COPD	5 (15.63%)	2 (8.33%)	0.683
Bronchiectasis	15 (46.88%)	6 (25.00%)	0.094
Pulmonary interstitial fibrosis	2 (6.25%)	7 (29.17%)	0.030^*^
Diabetes mellitus	4 (12.50%)	3 (12.50%)	1.000
Hypertension	9 (28.13%)	8 (33.33%)	0.675
Coronary heart diseases	9 (28.13%)	5 (20.83%)	0.533
Rheumatic immune diseases	0 (0.00%)	13 (54.17%)	0.000^***^
Hematologic malignancy	0 (0.00%)	1 (4.17%)	0.429
Active solid tumor	1 (4.17%)	5 (20.83%)	0.074
Organ transplantation	0 (0.00%)	1 (4.17%)	0.429
Radiological findings, n (%)			
Ground-glass opacities	25 (78.13%)	19 (79.17%)	0.444
Nodules	16 (50.00%)	12 (50.00%)	0.743
Air bronchial sign	5 (15.63%)	1 (4.17%)	0.383
Thickening of the bronchial wall	10 (31.25%)	1 (4.17%)	0.019^*^
Cavitation sign	5 (15.63%)	3 (12.50%)	1.000
Pleural thickening	9 (28.13%)	5 (20.83%)	0.657
Pleural effusion	14 (43.75%)	9 (37.50%)	0.836
Lymphadenopathy	13 (40.63%)	15 (62.50%)	0.046^*^
Laboratory findings			
WBC (10^9^/L)	12.38 ± 7.53	10.46 ± 3.90	0.262
Neutrophils (10^9^/L)	10.39 ± 7.33	8.64 ± 3.89	0.293
Lymphocytes(10^6^/L)	0.97(0.72-1.35)	1.00(0.58-1.95)	0.921
CRP (mg/ml)	58.27(6.14-142.13)	59.99(22.63-112.10)	0.900
ESR (mm/h)	60.32 ± 37.34	69.11 ± 29.14	0.408
PCT (ng/ml)	0.12(0.07-0.58)	0.14(0.05-0.35)	0.969

ICPs: immunocompetent patients; ISPs: immunosuppressed patients.

^*^ *P* < 0.05; ^**^P < 0.01, ^***^P < 0.001.

Among these ND cases, 24 (42.86%) identified *Nocardia* as the sole pathogen. The proportion of patients with mixed fungal infections was higher in the ISPs group than that in the ICPs group (54.17% vs. 18.75%, *P* = 0.006) (**Figure 4A**). Among those with *Nocardia* as the sole pathogen, *N. cyriacigeorgica* was significantly more prevalent in the ISPs group than in the ICP group (55.56% vs. 5.88%, *P* = 0.007) (**Figure 4B**). Additionally, the top 10 microbial species co-detected with *Nocardia* in ISPs and ICPs patients were shown in **Figure 4C**. Among them, only *Pneumocystis jirovecii* showed significant differences between the two groups (*P* < 0.001).

**Figure 4:**
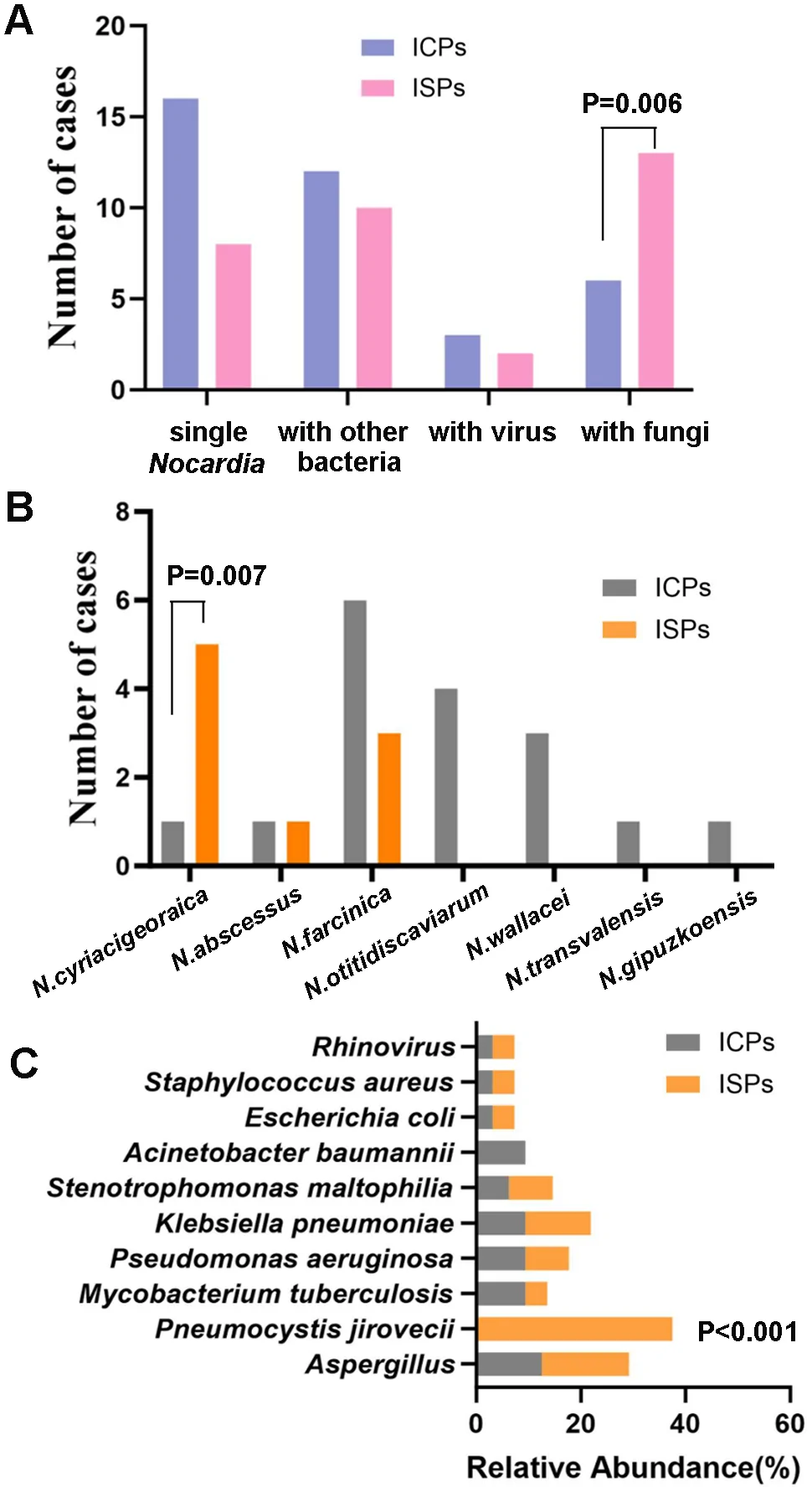
Differences in co-pathogens, single *Nocardia*, and top 10 species between ICPs and ISPs in ND patients. **(A)** Composition differences of co-pathogens between the two patient cohorts. The proportion of fungal co-infections was significantly higher in ISPs than in ICPs (P = 0.006). **(B)** Species distribution of single *Nocardia* infections stratified by immune status. *N. cyriacigeorgica* was significantly more prevalent in ISPs than in ICPs (P = 0.007). Other species showed no significant differences between groups. **(C)** Bar plot showed the top 10 species with the highest abundance between the two patient cohorts.

### Impacts of mNGS on antimicrobial usage of *Nocardia* infection patients

In our study, mNGS results were associated with a change in anti-*Nocardia* management in 49/56 patients (87.5%), including initiation of targeted therapy (n = 36) and regimen adjustment (n = 13) (**Table 4**). Antibiotic regimens were not adjusted for seven patients: two died of critical illness despite optimal supportive care; two continued empiric therapy effective against *Nocardia*; two with confirmed tuberculosis were referred to a specialized hospital; and one with advanced cancer received palliative treatment focused on the primary disease.

**Table 4:** Clinical impact of mNGS results on ND management.

Clinical impact	n (%)	Outcomes	n/N(%)	Relapse (≥6 M)
**Treatment changed**	49 (87.5%)			
Initiation of targeted anti-*Nocardia* therapy	36 (64.3%)	Improvement	34/36 (94.4%)	2/36 (5.6%)
Regimen adjustment	13 (23.2%)	Improvement	11/13 (84.6%)	1/13 (7.7%)
**Treatment not changed**	7 (12.5%)			
Continued effective empiric therapy	2 (3.6%)	Improvement	1/2 (50%)	0/2 (0%)
Death due to critical illness	2 (3.6%)	Death	_	_
Referred for tuberculosis Treatment	2 (3.6%)	Improved after anti-TB therapy	2/2 (100%)	_
Palliative care (advanced cancer)	1 (1.8%)	Improvement	1/1 (100%)	_

As shown in **Table 4**, among the 49 patients receiving mNGS-guided treatment modifications, clinical improvement was observed in 45 patients (91.8%), including 34/36 (94.4%) in the targeted therapy initiation group and 11/13 (84.6%) in the regimen adjustment group. The overall 28-day mortality in the ND group was 12.5% (7/56; **Table 1**), with two deaths occurring in patients who did not receive treatment modification due to rapid deterioration, and five deaths occurring despite treatment modification. Among patients with at least 6 months of follow-up, relapse occurred in 3 patients (3/49, 6.1%). Notably, all three relapses occurred exclusively in ISPs (3/22, 13.6%), while no relapse was documented in ICPs.

Comprehensive records of anti-*Nocardia* therapy for all ND patients have been systematically collected (**Table 5**). Among them, 2 patients (3.6%) received trimethoprim-sulfamethoxazole (TMP-SMZ) monotherapy; 43 patients (76.8%) received TMP-SMZ in combination with at least one other antibiotic. The adjunctive agents included oxazolidinones (linezolid), carbapenems (imipenem and meropenem), β-lactam/β-lactamase inhibitor combinations (cefoperazone-sulbactam), and fluoroquinolones (moxifloxacin and levofloxacin). The remaining 11 patients (19.6%) received alternative regimens that did not included TMP-SMZ.

**Table 5:** Antibiotic treatment for patients in the ND group.

Antibiotic therapy	Patients n (%)	Improve n (%)
Only TMP-SMZ	2 (3.6%)	2 (3.6%)
Combination		39 (69.64%)
+LZ	6 (10.7%)	
+LZ+MFX	1 (1.8%)	
+LZ+CPS	1 (1.8%)	
+LZ+IMP	10 (17.9%)	
+LZ+LEV	7 (12.5%)	
+CPS	4 (7.1%)	
+IMP	12 (21.4%)	
+IMP+MFX	1 (1.8%)	
+IMP+LEV	1 (1.8%)	
One or more antibiotics other than TMP-SMZ	11 (19.6%)	8 (14.28%)

TMP-SMZ, trimethoprim-sulfamethoxazole; IMP, imipenem; LZ, linezolid; LEV, levofloxacin; CPS, cefoperazone-sulbactam; MFX, moxifloxacin.

## Discussion

The gold standard for nocardiosis identification is the isolation and culture of *Nocardia (*Fatahi-Bafghi, 2018). Despite clear diagnostic criteria, timely and accurate diagnosis of *Nocardia* infection or colonization/contamination remains clinically complex and challenging. Recent studies have reported that a positive *Nocardia* culture does not inherently signify infection, as colonization/contamination remains a potential interpretation (Margalit et al., 2020; Serino et al., 2022). With the widespread application of mNGS, an increasing number of *Nocardia* infections have been detected (Li et al., 2023; Qi et al., 2023; Zhang et al., 2023; Wang et al., 2024). Although some studies have noted that mNGS detection of *Nocardia* may be confounded by colonization/contamination, no study has specifically focused on distinguishing *Nocardia* infection from colonization/contamination.

In the present study, 56 (75.7%) patients were ultimately clinically diagnosed with ND, while the remaining 18 (24.3%) were classified as non-ND. This proportion is consistent with previously reported rates of *Nocardia* colonization (Margalit et al., 2020). By the end of 2025, 173 *Nocardia* species have been formally validated, of which 54 have been isolated from human clinical specimens (Chen et al., 2025; Prieto et al., 2025). In our cohort, *N. farcinica*, *N. cyriacigeorgica*, and *N. abscessus* were the most prevalent species. This distribution is consistent with recent epidemiological data from China, where *N. farcinica* and *N. cyriacigeorgica* have been reported as the leading species (Wang et al., 2023; Li et al., 2026). The predominance of *N. farcinica* among *Nocardia* species is linked to its enhanced virulence and antimicrobial resistance, highlighting the necessity of species-level identification to guide appropriate treatment (Nathar et al., 2025).

Numerous studies have investigated the diagnostic ability of mNGS for *Nocardia* infection (Ding et al., 2021; Jiao et al., 2022; Liang et al., 2023), but there remains little research on the distinction of *Nocardia* infection and colonization/contamination. In our study, *Nocardia* RPM values were significantly higher in the ND group than in the non-ND group, suggesting that mNGS-detected RPM values may aid this distinction. This finding aligns with growing evidence that quantitative parameters based on mNGS, such as RPM or raw sequencing reads, can serve as important indicators for distinguishing true pathogen infection from colonization (Duan et al., 2025; Zhou et al., 2025; Chen et al., 2026). To establish a quantitative diagnostic threshold of *Nocardia*, we performed ROC analysis, yielding an AUC of 0.774 (95% CI, 0.654–0.895) and identifying an optimal RPM cutoff of 56. Using RPM ≥ 56 as the criterion for *Nocardia* infection, mNGS demonstrated a sensitivity of 73.21%, which was higher than that of culture (25.0%; *P* < 0.001) and smear (12.5%; *P* < 0.001), and a specificity of 83.33% for the final diagnosis. These findings suggest that quantitative mNGS with an appropriate RPM threshold may help distinguish *Nocardia* infection from colonization/contamination. In addition, bootstrap validation confirmed the stability of the RPM threshold, with a median cutoff of 57 and identical in-bag and out-of-bag AUC (0.774), suggesting no overfitting. Nonetheless, the 95% CI for the cutoff was wide (21–285), reflecting the limited sample size, especially the small non-ND group (n = 18). This underscores the exploratory nature of our cutoff and the need for external validation in larger cohorts to improve precision and confirm generalizability.

We also performed a subgroup ROC analysis in immunocompetent patients, which produced an AUC of 0.845 (95% CI, 0.722–0.968). Applying the overall threshold (RPM ≥ 56) to immunocompetent patients gave a sensitivity of 81.25% and specificity of 81.25%, comparable to those obtained with an immunocompetent-specific threshold (RPM ≥ 46; sensitivity of 87.5%, specificity of 75.0%). These results suggest that the overall-cohort RPM threshold can be reasonably applied to immunocompetent patients. Accordingly, we propose a gray-zone interpretation framework for immunocompetent patients, with RPM values of approximately 45–60. In the absence of standardized quantitative methods for mNGS, this approach provides practical guidance for clinicians in interpreting mNGS results.

In our study, univariate and multivariate logistic regression identified three factors significantly associated with the diagnosis of ND. Farmers are considered a high-risk occupational group for nocardiosis because of long-term exposure to contaminated soil and organic dust (Kupila et al., 2011). We also found farming exposure was more common in the ND group, which was consistent with the environmental epidemiology of *Nocardia*. Given the large agricultural population in China, our research findings provide local data that, to some extent, help identify populations at high exposure risk.

Immunosuppression and bronchiectasis are two other independent clinical factors associated with an increased risk of nocardiosis. Although nocardiosis typically occurs in individuals with impaired cellular immunity (Restrepo and Clark, 2019), recent studies have increasingly shown that it can also develop in immunocompetent individuals. Immunocompetent patients with chronic obstructive pulmonary disease (COPD) or bronchiectasis exhibit a substantially elevated risk of developing pulmonary nocardiosis (Yang et al., 2023; Han et al., 2024; Li et al., 2026). In this population, corticosteroid therapy is thought to damage the lower respiratory tract epithelium, thereby facilitating *Nocardia* colonization (Galar et al., 2021). Approximately 57.14% of patients diagnosed with *Nocardia* infection in our study were immunocompetent, aligning well with the 54% observed in a recent study (Li et al., 2026).

Due to the variable radiological appearance of nocardiosis, differentiating it from tuberculosis, tumors, and other fungal infections was often challenging (Li et al., 2026). In our study, radiological images showed that lymphadenopathy was more common in ISPs patients (62.50% vs. 40.63%, P = 0.046), whereas bronchial wall thickening was more frequently observed in ICPs patients. This difference may be closely related to the host’s immune response mechanisms against *Nocardia* infection. In immunocompetent hosts, activated macrophages and T cells can effectively recognize and eliminate *Nocardia*, thereby restricting local spread of infection (Beaman and Beaman, 1994). This effective immune surveillance mechanism may confine the infection mainly to the airways and peri bronchial tissues, thereby manifesting as characteristic changes of tracheobronchial involvement, such as bronchial wall thickening. In contrast, in immunocompromised hosts, defective T cell function impairs the intracellular killing of bacteria within the lymph nodes. Consequently, *Nocardia* may persist and proliferate locally, causing reactive or infectious lymphadenopathy (Bargehr et al., 2013). Further studies are needed to clarify the pathogenic mechanisms of *Nocardia* in hosts with different immune statuses.

Beyond the immediate diagnostic utility, the immune-based phenotyping observed in our study carries several translational implications that may inform treatment algorithms. First, the rate of fungal co-infection in the ISPs group was significantly higher than that in the ICPs group (54.17% vs. 18.75%, *P* = 0.006), which is consistent with previous studies (Huang et al., 2019; Jiao et al., 2022). This difference may be related to the overall impaired immune function in immunosuppressed patients, making them more susceptible to multiple opportunistic pathogens. It also suggests that empirical antifungal coverage should be considered more aggressively in ISPs with severe nocardiosis, particularly when patients do not respond promptly to anti-*Nocardia* therapy alone. Second, distinct radiological patterns (lymphadenopathy in ISPs versus bronchial wall thickening in ICPs) could guide diagnostic choices: for ISPs, endobronchial ultrasound-guided transbronchial needle aspiration (EBUS-TBNA) is preferred to confirm *Nocardia* infection and exclude malignancy or other opportunistic infections. Third, the predominance of *N. cyriacigeorgica* as a sole pathogen in ISPs underscores the prognostic and therapeutic value of species-level identification for guiding initial antibiotic choices. These immune-guided strategies await prospective validation but offer a framework for more personalized management of nocardiosis.

An increasing number of studies have demonstrated the practical value of mNGS in informing clinical decisions (Jiao et al., 2022; Liang et al., 2023; Chen et al., 2025). We also found mNGS results had a positive impact on treatment decisions in 87.5% (49/56) of ND patients, and all relapses occurred exclusively in ISPs (13.6% vs. 0%). This finding suggests that ISPs may require more prolonged antimicrobial therapy and closer post-treatment surveillance compared to ICPs (Margalit et al., 2021; Attias et al., 2025). Our relapse data, while limited by small numbers, support this differential approach and underscore the value of immune status-based phenotyping in individualizing treatment duration. Although 32.2% of *Nocardia* were resistant to three or more commonly used antibiotics (Li et al., 2026), and multidrug resistance rates among different *Nocardia* species ranging from 1% to 35% (Valdezate et al., 2017), TMP-SMX and β-lactam antibiotics remain standard treatments for nocardiosis (Welsh et al., 2013; Lafont et al., 2020; Margalit et al., 2021; Besteiro et al., 2023). The antimicrobial resistance mechanisms of *Nocardia* are species-specific and genomically complex, which directly affects treatment decisions. β-lactam resistance is predominantly mediated by class A β-lactamases: the FAR-1 gene, present in *N. farcinica*, confers high level resistance to penicillins and narrow-spectrum cephalosporins (Laurent et al., 1999), whereas the AST-1 gene is exclusively found in *N. cyriacigeorgica (*Di et al., 2025). Resistance to TMP-SMZ can arise through sul1 and sul2 genes (Valdezate et al., 2015; Song et al., 2025), as well as through mutations in dihydrofolate reductase (DHFR) and dihydropteroate synthase (DHPS) (Mehta et al., 2018). Aminoglycoside resistance can be conferred by the 16S rRNA methyltransferase warA, which mediates pan-aminoglycoside resistance in *N. wallacei* (Hershko et al., 2024), while fluoroquinolone resistance is primarily driven by gyrA mutations (Song et al., 2025). In contrast, resistance to linezolid remains exceedingly rare among clinical *Nocardia* isolates.

These findings have direct stewardship implications. Given that linezolid demonstrates near-universal activity against *Nocardia* and TMP-SMZ remains highly susceptible in most species (Wang et al., 2023), we advocate TMP-SMZ as first line therapy for non-severe disease, while reserving linezolid for severe, disseminated, or central nervous system(CNS) infections. In addition, species level identification should be obtained to guide definitive therapy. Finally, the duration of treatment should be adjusted according to the immune status (Margalit et al., 2021). This immune-based stratification aligns with our observed higher relapse rate in ISPs and provides a practical framework for integrating mNGS results into individualized antimicrobial management.

With meticulously designed and analyzed, this study also exists limitations. First, this was a single-center retrospective study with a small sample size. The optimal RPM threshold requires external validation in larger, multicenter cohorts before it can be recommended for routine clinical use. Second, the absence of long-term follow-up data limits assessment of prognosis and treatment guidance. Third, although we incorporated host risk factors and a quantitative mNGS threshold, the interplay between RPM values and host immune status in predicting clinical outcomes warrants further investigation in prospective studies. Forth, although reagent-derived or cross-contamination cannot be fully excluded, all mNGS runs included negative controls and ambiguous signals were reviewed. Future studies using quantitative PCR or strain verification are needed. Despite these limitations, our findings offer practical insights for interpreting positive mNGS results of *Nocardia* in clinical practice.

In this retrospective study, we found that farming exposure, bronchiectasis, and immunocompromised status were significantly associated with true nocardiosis. An RPM threshold of 56 provided fair discrimination between infection and colonization/contamination. In addition, patients with different immune statuses exhibited distinct radiological and microbiological profiles. *Nocardia* was the sole pathogen in 42.9% of true infection cases, and mNGS results led to a change in anti-*Nocardia* management in 87.5% of patients. Our findings suggest that integrating host risk factors with a quantitative mNGS threshold (RPM ≥56) may improve the clinical interpretation of positive *Nocardia* results, and immune-status-based phenotyping may guide more individualized management.

## Data Availability

The datasets presented in this study can be found in online repositories. The raw sequence data reported in this paper have been deposited in the Genome Sequence Archive (Genomics, Proteomics & Bioinformatics 2025) in National Genomics Data Center (Nucleic Acids Res 2025), China National Center for Bioinformation / Beijing Institute of Genomics, Chinese Academy of Sciences (GSA: CRA039831) that are publicly accessible at https://ngdc.cncb.ac.cn/gsa.

## References

[B1] Attias N. H. Schlaeffer-Yosef T. Zahavi I. Hasson N. Ari Y. B. Darawsha B. . (2025). Shorter vs. standard-duration antibiotic therapy for nocardiosis: a multi-center retrospective cohort study. Infection 53, 1115–1127. doi: 10.1007/s15010-024-02445-0 39589427 PMC12137364

[B2] Bargehr J. Flors L. Leiva-Salinas C. Flohr T. R. Sawyer R. Bonatti H. . (2013). Nocardiosis in solid-organ transplant recipients: spectrum of imaging findings. Clin. Radiol. 68, e266-271. doi: 10.1016/j.crad.2012.11.021 23351777 PMC3816150

[B3] Beaman B. L. Beaman L. (1994). Nocardia species: host-parasite relationships. Clin. Microbiol. Rev. 7, 213–264. doi: 10.1128/cmr.7.2.213 8055469 PMC358319

[B4] Besteiro B. Coutinho D. Fragoso J. Figueiredo C. Nunes S. Azevedo C. . (2023). Nocardiosis: a single-center experience and literature review. Braz. J. Infect. Dis. 27, 102806. doi: 10.1016/j.bjid.2023.102806 37802128 PMC10582834

[B5] Brown-Elliott B. A. Brown J. M. Conville P. S. Wallace R. J. Jr. (2006). Clinical and laboratory features of the nocardia spp. based on current molecular taxonomy. Clin. Microbiol. Rev. 19, 259–282. doi: 10.1128/jcm.29.11.2407-2411.1991 16614249 PMC1471991

[B6] Chen J. Zhou H. Xu P. Zhang P. Ma S. Zhou J. (2014). Clinical and radiographic characteristics of pulmonary nocardiosis: clues to earlier diagnosis. PloS One 9, e90724. doi: 10.1371/journal.pone.0090724 24594890 PMC3940923

[B7] Chen Y. Fu H. Zhu Q. Ren Y. Liu J. Wu Y. . (2025). Clinical features of pulmonary nocardiosis and diagnostic value of metagenomic next-generation sequencing: a retrospective study. Pathogens 14, 656. doi: 10.3390/pathogens14070656 40732703 PMC12301029

[B8] Chen Y. Tang X. Lu S. Guo L. Wang L. Min L. . (2026). The diagnostic and prognostic utility of blood metagenomic next-generation sequencing for invasive pulmonary aspergillosis. Microbiol. Spectr. 14, e0338425. doi: 10.1128/spectrum.03384-25 41995327 PMC13228001

[B9] Coussement J. Lebeaux D. van Delden C. Guillot H. Freund R. Marbus S. . (2016). Nocardia infection in solid organ transplant recipients: A multicenter european case-control study. Clin. Infect. Dis. 63, 338–345. doi: 10.1093/cid/ciw241 27090987

[B10] Di L. Xu A. Li Y. Gu C. Xu H. Xia X. (2025). Genomic diversity of nocardia cyriacigeorgica and nocardia farcinica infections. Microb. Pathog. 205, 107602. doi: 10.1016/j.micpath.2025.107602 40280503

[B11] Ding J. Ma B. Wei X. Li Y. (2021). Detection of nocardia by 16S ribosomal RNA gene PCR and metagenomic next-generation sequencing (mNGS). Front. Cell. Infect. Microbiol. 11, 768613. doi: 10.3389/fcimb.2021.768613 35071035 PMC8779735

[B12] Duan Y. Li Q. Fei H. Song J. Xu C. (2025). The diagnostic value of RNA-mNGS and DNA-mNGS in differentiating bacterial infection from colonization in the lower respiratory tract. Front. Cell. Infect. Microbiol. 15, 1639148. doi: 10.3389/fcimb.2025.1639148 40995224 PMC12454375

[B13] Fatahi-Bafghi M. (2018). Nocardiosis from 1888 to 2017. Microb. Pathog. 114, 369–384. doi: 10.1016/j.micpath.2017.11.012 29146497

[B14] Galar A. Martín-Rabadán P. Marín M. Cercenado E. Sánchez-Carrillo C. Valerio M. . (2021). Revisiting nocardiosis at a tertiary care institution: Any change in recent years? Int. J. Infect. Dis. 102, 446–454. doi: 10.1016/j.ijid.2020.10.087 33157288

[B15] Han Y. Cheng M. Li Z. Chen H. Xia S. Zhao Y. . (2024). Clinical characteristics and drug resistance of nocardia in henan, China, 2017-2023. Ann. Clin. Microbiol. Antimicrob. 23, 23. doi: 10.1186/s12941-024-00677-4 38449006 PMC10919010

[B16] Hershko Y. Rannon E. Adler A. Burstein D. Barkan D. (2024). WarA, a remote homolog of NpmA and KamB from nocardia wallacei, confers broad spectrum aminoglycoside resistance in nocardia and mycobacteria. Int. J. Antimicrob. Agents 63, 107089. doi: 10.1016/j.ijantimicag.2024.107089 38218322

[B17] Hill A. T. (2020). Management of community-acquired pneumonia in immunocompromised adults: A consensus statement regarding initial strategies. Chest 158, 1802–1803. doi: 10.1016/j.chest.2020.08.003 33160518

[B18] Huang L. Sun L. Yan Y. (2019). Characteristics of nocardiosis patients with different immune status from a chinese tertiary general hospital during 8-year period: A STROBE-compliment observational study. Med. (Baltimore). 98, e17913. doi: 10.21203/rs.2.16153/v1 31702669 PMC6855654

[B19] Jiao M. Ma X. Li Y. Wang H. Liu Y. Guo W. . (2022). Metagenomic next-generation sequencing provides prognostic warning by identifying mixed infections in nocardiosis. Front. Cell. Infect. Microbiol. 12, 894678. doi: 10.3389/fcimb.2022.894678 36118026 PMC9471186

[B20] Kandi V. (2015). Human nocardia infections: A review of pulmonary nocardiosis. Cureus 7, e304. doi: 10.7759/cureus.304 26430578 PMC4571773

[B21] Kupila N. Kankaanranta H. Sauni R. (2011). pulmonary nocardiosis in a farmer. Duodecim 127, 2315–2319. 22204147

[B22] Lafont E. Conan P. L. Rodriguez-Nava V. Lebeaux D. (2020). Invasive nocardiosis: disease presentation, diagnosis and treatment - old questions, new answers? Infect. Drug Resist. 13, 4601–4613. doi: 10.2147/idr.s249761 33376366 PMC7764858

[B23] Laurent F. Poirel L. Naas T. Chaibi E. B. Labia R. Boiron P. . (1999). Biochemical-genetic analysis and distribution of FAR-1, a class A beta-lactamase from nocardia farcinica. Antimicrob. Agents Chemother. 43, 1644–1650. doi: 10.1128/AAC.43.7.1644 PMC89337 10390216

[B24] Li X. Feng Y. Li D. Chen L. Shen M. Li H. . (2023). Cerebral abscess infected by nocardia gipuzkoensis. Infect. Drug Resist. 16, 7247–7253. doi: 10.2147/idr.s428415 38023399 PMC10657549

[B25] Li X. Liu C. Hu Y. Xu H. Li H. Sun J. . (2026). Epidemiology, clinical treatment and outcomes, susceptibility patterns and genotypic analysis of 214 nocardia strains from multiple centers in henan province. Front. Cell. Infect. Microbiol. 16, 1728269. doi: 10.3389/fcimb.2026.1728269 41769349 PMC12945825

[B26] Liang Y. Lin M. Qiu L. Chen M. Tan C. Tu C. . (2023). Clinical characteristics of hospitalized patients with nocardia genus detection by metagenomic next generation sequencing in a tertiary hospital from southern China. BMC Infect. Dis. 23, 772. doi: 10.1186/s12879-023-08615-z 37940842 PMC10634012

[B27] Margalit I. Lebeaux D. Tishler O. Goldberg E. Bishara J. Yahav D. . (2021). How do I manage nocardiosis? Clin. Microbiol. Infect. 27, 550–558. doi: 10.1016/j.cmi.2020.12.019 33418019

[B28] Margalit I. Muhsen K. Ben Ari Y. Ben-Zvi H. Shostak Y. Krause I. . (2020). Nocardia colonization in contrast to nocardiosis: a comparison of patients' clinical characteristics. Eur. J. Clin. Microbiol. Infect. Dis. 39, 759–763. doi: 10.1007/s10096-019-03796-5 31863237

[B29] Mehta H. Weng J. Prater A. Elworth R. A. L. Han X. Shamoo Y. (2018). Pathogenic nocardia cyriacigeorgica and nocardia nova evolve to resist trimethoprim-sulfamethoxazole by both expected and unexpected pathways. Antimicrob. Agents Chemother. 62, e00364-18. doi: 10.1128/aac.00364-18 29686152 PMC6021631

[B30] Miao Q. Ma Y. Wang Q. Pan J. Zhang Y. Jin W. . (2018). Microbiological diagnostic performance of metagenomic next-generation sequencing when applied to clinical practice. Clin. Infect. Dis. 67, S231–S240. doi: 10.1093/cid/ciy693 30423048

[B31] Nathar S. Nagarajan H. Narthanareeswaran B. Pandian C. J. Thiyonila B. Jeyaraman J. (2025). In silico identification of novel alpha/beta hydrolase fold domain-containing protein associated with virulence and antibiotic resistance in nocardia farcinica (strain: JJSBBCNF_01). Mol. Divers. 30, 5207–5225. doi: 10.1007/s11030-025-11415-z 41288902

[B32] Prieto M. Rocca M. F. Armitano R. Gianecini A. Cipolla L. (2025). Antimicrobial resistance patterns of nocardia species in clinical isolates from Argentina (2020-2022). Enferm. Infecc. Microbiol. Clin. (Engl. Ed). 43, 659–665. doi: 10.1016/j.eimc.2025.07.003 41338821

[B33] Qi D. D. Zhuang Y. Chen Y. Guo J. J. Zhang Z. Gu Y. (2023). Interstitial pneumonia combined with nocardia cyriacigeorgica infection: A case report. World J. Clin. cases 11, 7920–7925. doi: 10.12998/wjcc.v11.i32.7920 38073689 PMC10698421

[B34] Restrepo A. Clark N. M. (2019). Nocardia infections in solid organ transplantation: Guidelines from the infectious diseases community of practice of the american society of transplantation. Clin. Transplant. 33, e13509. doi: 10.1111/ctr.13509 30817024

[B35] Serino M. Sousa C. Redondo M. Carvalho T. Ribeiro M. Ramos A. . (2022). Nocardia spp. isolation in chronic lung diseases: Are there differences between patients with pulmonary nocardiosis and nocardia colonization? J. Appl. Microbiol. 133, 3239–3249. doi: 10.1111/jam.15778 35957549

[B36] Song Z. Du B. Yuan M. Shen J. Xu S. Guan W. . (2025). Genomic and phenotypic characterization of antimicrobial resistance in clinical nocardia species isolates. Front. Cell. Infect. Microbiol. 15, 1672889. doi: 10.3389/fcimb.2025.1672889 41050760 PMC12488653

[B37] Valdezate S. Garrido N. Carrasco G. Medina-Pascual M. J. Villalón P. Navarro A. M. . (2017). Epidemiology and susceptibility to antimicrobial agents of the main nocardia species in Spain. J. Antimicrob. Chemother. 72, 754–761. doi: 10.1093/jac/dkw489 27999029

[B38] Valdezate S. Garrido N. Carrasco G. Villalón P. Medina-Pascual M. J. Saéz-Nieto J. A. (2015). Resistance gene pool to co-trimoxazole in non-susceptible nocardia strains. Front. Microbiol. 6, 376. doi: 10.3389/fmicb.2015.00376 25972856 PMC4412068

[B39] Verfaillie L. De Regt J. De Bel A. Vincken W. (2010). Nocardia asiatica visiting Belgium: nocardiosis in a immunocompetent patient. Acta Clin. Belg. 65, 425–427. doi: 10.1179/acb.2010.65.6.010 21268959

[B40] Wang C. Sun Q. Yan J. Liao X. Long S. Zheng M. . (2023). The species distribution and antimicrobial resistance profiles of nocardia species in China: a systematic review and meta-analysis. PloS Negl.Trop. Dis. 17, e0011432. doi: 10.1371/journal.pntd.0011432 37428800 PMC10358964

[B41] Wang D. Hu M. T. Liu W. J. Zhao Y. Xu Y. C. (2024). Bacteremia caused by nocardia farcinica: a case report and literature review. BMC Infect. Dis. 24, 381. doi: 10.1186/s12879-024-09230-2 38589778 PMC11003049

[B42] Welsh O. Vera-Cabrera L. Salinas-Carmona M. C. (2013). Current treatment for nocardia infections. Expert Opin. Pharmacother. 14, 2387–2398. doi: 10.1517/14656566.2013.842553 24093436

[B43] Weng S. S. Zhang H. Y. Ai J. W. Gao Y. Liu Y. Y. Xu B. . (2020). Rapid detection of nocardia by next-generation sequencing. Front. Cell. Infect. Microbiol. 10, 13. doi: 10.3389/fcimb.2020.00013 32133300 PMC7040243

[B44] Yang J. Ren H. T. Wang J. Dong A. Y. Chen Y. L. Hu D. X. . (2023). Clinical characteristics, susceptibility profiles, and treatment of nocardiosis: a multicenter retrospective study in 2015-2021. Int. J. Infect. Dis. 130, 136–143. doi: 10.1016/j.ijid.2023.02.023 36871785

[B45] Zhang J. Zhu Y. Sun Y. Han X. Mao Y. (2023). Pathogenic nocardia amamiensis infection: A rare case report and literature review. Heliyon 9, e17183. doi: 10.1016/j.heliyon.2023.e17183 37449159 PMC10336398

[B46] Zhou X. Yang C. Liu X. Wang J. Li Y. Pan L. . (2025). Clinical performance of metagenomic next-generation sequencing for distinction and diagnosis of mucorales infection and colonization. Front. Cell. Infect. Microbiol. 15, 1631960. doi: 10.3389/fcimb.2025.1631960 41050761 PMC12488623

