# Supplementary material for: When metagenomic next-generation sequencing detects Nocardia: from clinical interpretation to immune-based clinical management
Source: Front Cell Infect Microbiol. 2026 Sep 18;16:1911562. doi: 10.3389/fcimb.2026.1911562 (PMC13630650; doi:10.3389/fcimb.2026.1911562)
Supplement: Supplementary file 1 [file SupplementaryFile1.docx]

**Appendix 1.**

**Table S1** Follow-up outcomes of the 18 patients initially classified as non-ND

| **Patient No.** | **Follow-up (days)**） | **Follow-up method** | **Outcome** |
| --- | --- | --- | --- |
| **1** | 137 | Telephone interview | Alive, no evidence of nocardiosis |
| **2** | 298 | Telephone interview | Alive, no evidence of nocardiosis |
| **3** | 569 | Telephone interview | Alive, no evidence of nocardiosis |
| **4** | - | Lost to follow-up | Not assessable |
| **5** | 179 | Telephone interview | Alive, no evidence of nocardiosis |
| **6** | 55 | Hospitalization at another facility (fever) | Nocardiosis not diagnosed |
| **7** | 59 | Hospitalization at another facility (fever) | Nocardiosis not diagnosed |
| **8** | 13 | Telephone interview | **Died** ( unrelated to nocardiosis) |
| **9** | 90 | Telephone interview | Alive, no evidence of nocardiosis |
| **10** | 58 | Outpatient revisit | Symptoms resolved, CT not repeated |
| **11** | 27 | Telephone interview | Alive, no evidence of nocardiosis |
| **12** | 257 | Telephone interview | Alive, no evidence of nocardiosis |
| **13** | 412 | Telephone interview | Alive, no evidence of nocardiosis |
| **14** | 44 | Telephone interview | Alive, no evidence of nocardiosis |
| **15** | 412 | Medical records | Nocardiosis not diagnosed |
| **16** | 157 | Outpatient revisit | Symptoms resolved, CT not repeated |
| **17** | 812 | Telephone interview | Alive, no evidence of nocardiosis |
| **18** | 150 | Outpatient revisit (CT) | CT improved, no recurrence |

**Appendix 2.**

**Detailed Procedures of Metagenomic Next-generation Sequencing**

**Sample Preprocessing**

Sample preprocessing is critical to minimize host DNA interference and enhance pathogen detection sensitivity. All clinical specimens, including blood, cerebrospinal fluid (CSF), bronchoalveolar lavage fluid (BALF) and abscess, were collected aseptically and processed immediately or stored properly until use. For blood, centrifugation was applied to separate cellular components: 1,600g for 10 minutes to pellet host cells, followed by collection of the supernatant for downstream processing. For BALF, purulent exudate and other sticky samples were pre-treated with trypsin (final concetration 1%).

To minimize host DNA contamination of BALF and sputum samples, differential lysis strategies were employed. Hypotonic lysis was used to selectively lyse host cells while preserving microbial integrity. After benzonase treatment, samples (except plasma) were subjected to mechanical disruption using bead beating (0.1-1 mm mixed silica beads, 6,000 rpm for 10 minutes) was optimized to enhance cell wall penetration.

**Nucleic Acid Purification**

DNA was extracted and purified using a magnetic bead-based method with nucleic acid extraction kits (VM031-50, Visionmedicals) to ensure high-purity DNA recovery from low-biomass samples. Briefly, 250 μL of processed sample was mixed with lysis buffer and 20 μL proteinase K. The mixture underwent lysis at 70°C for 10 minutes, followed by magnetic separation to bind nucleic acids. Sequential washing steps (800 μL of low-salt and high-salt buffers) removed contaminants, and nucleic acids were eluted in 60–120 μL nuclease-free water.

**Construction of DNA library**

The extracted DNA samples were used to construct DNA Libraries. Purification and size selection were carried out following the double-sided bead purification procedure. A Qubit dsDNA HS assay kit (Invitrogen, USA) was used to measure the library concentration with an Invitrogen™ Qubit™ 4 Fluorometer. The library was prepared by pooling a 1.5 pM concentration of each purified sample equally for sequencing on an MGISEQ 200 sequencer using a 50-cycle single-end sequencing strategy.

**Bioinformatics analyses**

For bioinformatics analysis, trimmomatic was used to remove low-quality reads, adapter contamination and duplicate reads^(1)^. Low-complexity reads were removed by K-complexity using default parameters. The human sequence data were identified and excluded by mapping to a human reference genome (hg38) using SNAP v1.0beta.18^(2)^.

To construct the microbial genome database, pathogens and their genomes or assemblies were selected following the Kraken2 criteria for selecting representative assemblies for microorganisms from the NCBI Assembly and Genome databases (https://benlangmead.github.io/aws-indexes/k2). Microbial reads were aligned to the database using Burrows-Wheeler Aligner software^(3)^. We defined that reads with 90% identity of reference were mapped reads. In addition, reads with multiple locus alignments within the same genus were excluded from the secondary analysis. Only reads mapped to the genome within the same species were considered.

**Interpretation of metagenomic data and an official report to the clinician**

We normalized the sequencing reads RPM to eliminate the errors caused by various sequencing depths among samples. To establish the optimal threshold value for the >10 microbes with culture isolates, samples spiked with microbes were defined as positive samples, while negative control (NC) was defined as the negative sample. Receiver operating characteristic curves were plotted for each target species using these samples. The parameter resulting in the highest area AUC was considered the positive cutoff value for this species^(4)^. For microorganisms without culture isolates, the RPM mean value and standard deviation of this microorganism were calculated, and the RPM (mean + 2 standard deviations [SD]) was set as a positive cutoff value ^(5)^.

The clinical reportable range (CRR) for pathogens was established according to the following three references indicated in a previous study^(5)^: (i) the Johns Hopkins ABX Guide (https://www.hopkinsguides.com/hopkins/index/Johns_Hopkins_ABX_Guide/Pathogens), (ii) Manual of Clinical Microbiology^(6)^, and (iii) clinical case reports or research articles published in peer-reviewed journals. All microbes that exceeded the threshold of mNGS were classified into 3 categories^(7)^: (i) probable (BALF mNGS-based results were within the CRR and concordant with the clinical and radiologic results; the RPTM was significantly higher than the positive cutoff value, and the abundance was obviously higher than that of other species of the same genus), (ii) possible (the microbe has pathogenic potential, but an alternate explanation is more likely), and (iii) unlikely (the microbe cannot cause pneumonia).

To monitor the sources of potential contamination, both NC and sterile deionized water, which served as nontemplate controls, were prepared in parallel with other samples in each batch^(8)^. In addition, we used sterile cotton swabs dipped in sterile deionized water to wipe the surfaces of the centrifuge and biosafety cabinet to generate the background microorganism list in our laboratory.

**Reference**

(1). Bolger AM, Lohse M, Usadel B. 2014. Trimmomatic: a flexible trimmer for Illumina sequence data. Bioinformatics 30:2114–2120. doi:10.1093/bioinformatics/btu170.

(2). Zaharia M, Bolosky WJ, Curtis K, Fox A, Patterson D, Shenker S, Stoica I, Karp RM, Sittler T. 2011. Faster and more accurate sequence alignment with SNAP. arXiv 1111.5572 [cs.DS]. https://arxiv.org/abs/1111.5572.

(3). Li H, Durbin R. 2009. Fast and accurate short read alignment with Burrows-Wheeler transform. Bioinformatics 25:1754–1760.doi: 10.1093/bioinformatics/btp324.

(4). Unal I. 2017. Defining an optimal cut-point value in ROC analysis: an alternative approach. Comput Math Methods Med 2017:3762651. doi: 10.1155/2017/3762651.

(5). Jing C, Chen H, Liang Y, Zhong Y, Wang Q, Li L, Sun S, Guo Y, Wang R, Jiang Z, Wang H. 2021. Clinical evaluation of an improved metagenomic next-generation sequencing test for the diagnosis of bloodstream infections. Clin Chem 67:1133–1143. doi: 10.1093/clinchem/hvab061.

(6). Jorgensen JH, Carroll KC, Funke G, Pfaller MA, Landry ML, Richter SS, Warnock DW (ed). 2015. Manual of clinical microbiology, 11th ed. ASM Press, Washington, DC.

(7). Chen H, Yin Y, Gao H, Guo Y, Dong Z, Wang X, Zhang Y, Yang S, Peng Q, Liu Y, Wang H. 2020. Clinical utility of in-house metagenomic next-generation sequencing for the diagnosis of lower respiratory tract infections and analysis of the host immune response. Clin Infect Dis 71:416–426.

(8). Miller S, Naccache SN, Samayoa E, Messacar K, Arevalo S, Federman S, Stryke D, Pham E, Fung B, Bolosky WJ, Ingebrigtsen D, Lorizio W, Paff SM, Leake JA, Pesano R, DeBiasi R, Dominguez S, Chiu CY. 2019. Laboratory validation of a clinical metagenomic sequencing assay for pathogen detection in cerebrospinal fluid. Genome Res 29:831–842. doi: 10.1101/gr.238170.118.
